# Comprehensive climatic suitability evaluation of peanut in Huang-Huai-Hai region under the background of climate change

**DOI:** 10.1038/s41598-022-15465-3

**Published:** 2022-07-05

**Authors:** Sicheng Wei, Kaiwei Li, Yueting Yang, Chunyi Wang, Cong Liu, Jiquan Zhang

**Affiliations:** 1 School of Environment, Northeast Normal University, Changchun, 130024 China; 2 Key Laboratory for Vegetation Ecology, Ministry of Education, Changchun, 130117 China; 3 State Environmental Protection Key Laboratory of Wetland Ecology and Vegetation Restoration, Northeast Normal University, Changchun, 130117 China; 4 Chinese Academy of Meteorological Sciences, Beijing, 100081 China

**Keywords:** Climate sciences, Ecology

## Abstract

The climate changes influence the growing suitability of peanut, an important oil crop. Climatic suitability evaluation in the Huang-Huai-Hai region, the main peanut producing region of China, which can optimize peanut planting structure and provide basis for increasing output. In this study, the temperature, precipitation, sunshine and comprehensive suitability models were established by using the climatic suitability function in different growth periods of peanut. In this study, the climate suitability function of peanut in different growth periods was used to establish the temperature, precipitation, sunshine and comprehensive suitability model. Combined with the meteorological data after Anusplin interpolation, the spatial distribution and chronological change of peanut climate suitability were analyzed. The results show that with climate change, the overall climate becomes warmer and drier and the temperature and precipitation suitability increase, but the sunshine suitability decreases. Based on the comprehensive suitability model, the suitability evaluation results are divided into four levels: the most suitable, suitable, sub-suitable and unsuitable. Among them, the most suitable peanut planting areas in the Huang-Huai-Hai region are concentrated in the west of the Haihe River Basin and the Huaihe River Basin. The data from the next 30 years show that both the most suitable and suitable areas have been expanded. Through the verification of yield correlation analysis and spatial distribution of disaster frequency, it can be seen that the evaluation results have high accuracy, which can be used to guide and optimize peanut production practices.

## Introduction

Climate change has caused disruptions in climatic resources such as global temperature and precipitation patterns that have lasted for tens of millions of years on the earth, severely impacting the global ecological and biological growth systems on which human societies depend, and has become an important factor affecting the safety of human crop^1–3^. The sixth assessment report of the United Nations IPCC shows that the impact of climate change is unprecedented and irreversible. In the next 100 years, the global average temperature may rise by 1–3.5 °C^4^. This kind of climate change will result in huge changes in space, time and amount of climatic resources, which will also have a great impact on the global crop planting system and layout.

Peanuts are important oilseeds and economic crops that can be used for food, oil, or exported for foreign exchange. Due to its wide range of uses peanuts have become an important strategic reserve crop for the sustainable development of China's agriculture in the future. It is also one of the leading crops that promote the sustainable development in the country's agriculture^5,6^. Peanuts are sensitive to the conditions of growth and development, and their yield and quality are closely related to the climatic resources such as temperature, precipitation and sunshine in the planting area^7^. The Huang-Huai-Hai region is one of the major peanut production bases in China. The sown area and output of peanuts account for approximately 60% of the nation’s total, occupying a pivotal position. However, global climate change has led to an increase in heat resources, bringing a shift in agro-climatic zones, and a prolonged crop growing season^8^, both of which have had an important impact on the peanut planting system and layout^9^. The sowing area of peanut in Huang-Huai-Hai region increased from 6.3 × 10^5^ hm^2^ in 1960 to 2.4 × 10^6^ hm^2^ in 2019, with an increase of 280%, and the yield increased from 4.3 × 10^3^ kg/hm^5^ in 1960 to 2.8 × 10^4^ kg/hm^2^ in 2019, with an increase of 574%. Therefore, the climatic suitability evaluation of peanuts in the Huang-Huai-Hai region under the background of climate change will guide the scientific and rational layout of peanut planting, avoid the impact of unfavorable meteorological disasters, and promote the sustainable development of the peanut industry.

Agro-climatic zonings are produced because of the needs of agricultural production. Based on agricultural climatic analysis, following the distribution law of agricultural climatic, applying some decisive climatic indexes, referring to human conditions, topographic and geomorphic conditions and other indicators, to divide large-scale or a particular research area into space areas or types, and then estimate the trend of climatic factors on agricultural cultivation and crop growth and development. At present, the widely used methods of climatic zoning include the following three types: First, a comprehensive analysis of crops' growth characteristics allows the key factors affecting their production to be considered as zoning indicators. Wang et al. classified the climatic region of wine grape in Ningxia with the effective cumulative temperature index^10^. Zhao et al. selected the accumulated temperature ≥ 10 °C significantly related to the average yield index as the zoning index according to the cultivation mode, determined the appropriate threshold under the dynamic cultivation mode by using the yield data, and evaluated the climatic suitability by using a fuzzy suitability method^11^. Secondly, the maximum entropy model (MaxEnt) is used to determine the maximum entropy of species probability distribution from the existing distribution data and environment, to predict the distribution model of species, and to perform zoning analysis combined with GIS technology. On the one hand, Wang et al. used MaxEnt model to evaluate the changes of potato climatic suitability in China from 1961 to 2017 and developed reasonable zoning standards for potato suitability zones^12^. Yao et al. used MaxEnt model to predict the distribution and suitability of global wheat planting potential under various global climatic change scenarios based on a large number of occurrence point data of wheat as well as the data of main environmental factors affecting wheat growth^13^. Thirdly, the suitability for crop growth and development is characterized by the quantitative changes of climatic factors such as temperature, precipitation and sunshine. Furthermore, the zoned area should be considered by its comprehensive suitability. For example, Yang et al. evaluated the climatic suitability change of spring maize planting area in Inner Mongolia based on the comprehensive suitability model and variety layout, and investigated the quantitative and inter-annual relationship between regional climatic suitability and meteorological yield by using stepwise regression and cross wavelet transform^14^. Tang et al. analyzed water, temperature and solar radiation using agro-climatic suitability theory and fuzzy mathematics method, and combined this with future climatic data, analyzed the characteristics of suitable areas of wheat and maize in North China Plain in the context of climate change^15,16^.

However, most of the previous studies have focused on food crops and characteristic forest and fruit crops^17–22^, few studies have been conducted on the growth suitability zoning of peanut, a field economic crop. Cui et al. mathematically divided Shandong province into five peanut type zones according to the growth pattern of peanuts^23^. Li et al. defined the quality of peanuts into zones^24^. Wang classified Liaoning peanuts into zones based on GIS technology^25^. Yang et al. obtained the climatic dominant area of peanut cultivation in China by using GIS technology^26^. In summary, most of the current studies on peanut climatic suitability zoning suffer from limitations such as short time series, small study ranges, single selection index and no distinction between reproductive stages. On the basis of the existing research, the temperature, precipitation and sunshine conditions for peanut growth and development in the Huang-Huai-Hai region have been thoroughly considered. A comprehensive suitability model for peanut growing season in the Huang-Huai-Hai region was built by considering temperature, precipitation and sunshine suitability of each growth period, in addition the spatial distribution and chronological variation characteristics of peanut climatic suitability in different climatic years were analyzed, which can provide scientific basis for rational utilization of climatic resources and adjustment of planting techniques, this will also increase peanut production income, and help economic development.

## Material and methods

### Overview of the study area

**Figure 1:**
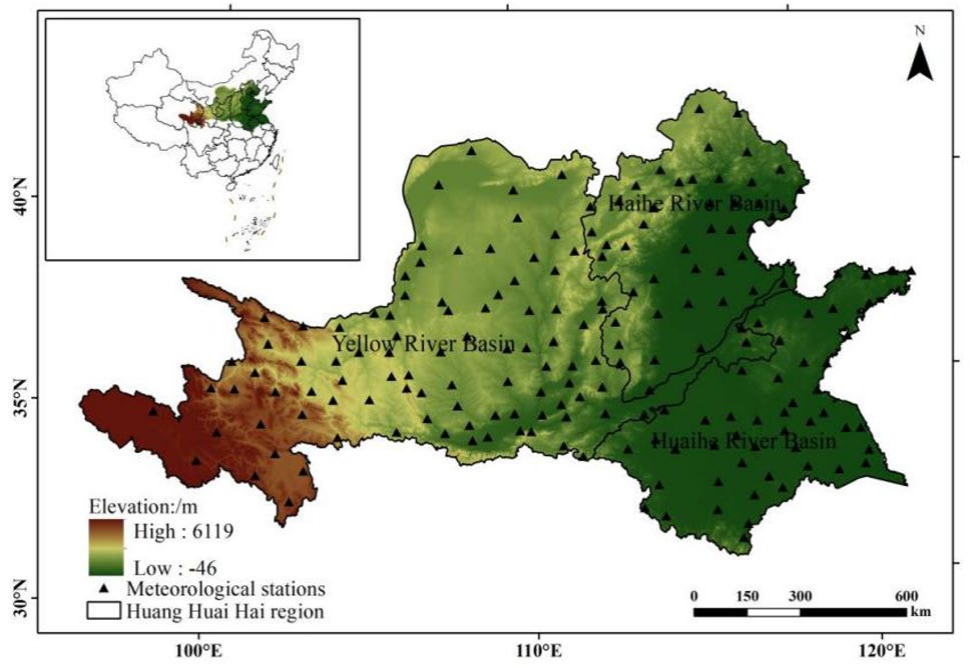
Location of the study areas. The figure was made in the ArcGIS 10.2 platform (https://www.esri.com/en-us/home).

Based on the actual cultivation of peanuts, the Huang-Huai-Hai region is selected as the study area (Fig. 1). The main body of the study area is the Huang-Huai-Hai Plain (North China Plain), which is a typical alluvial plain resulting from extensive sediment deposition carried by the Yellow River, the Huaihe River and the Haihe River and their tributaries, and the hills in central and southern Shandong Peninsula adjacent to it. Administrative zones include 5 provinces, 2 cities, 53 cities and 376 counties (districts). In China, The Huang-Huai-Hai region is an important production and processing centre for agricultural products, with a total land area of 4.10 × 10^5^ square kilometers and cultivated fields of 2.15 × 10^7^ hm^2^, accounting for 4.3% and 16.3% of the total amount of the country, respectively. It belongs to temperate continental monsoon climate with distinct seasons, accumulated temperature of 3600–4800 degrees above 10 °C, frost-free period of 170–200 days and annual precipitation of 500–950 mm^27^. The Huang-huai-hai region is the largest peanut growing area, accounting for more than 50% of the country's peanut production and area^28^.

### Data sources

The data used in the study mainly include meteorological data, geographic information data and crop data. The meteorological data comes from China Meteorological Information Center (http://data.cma.cn), including the daily maximum temperature (℃), daily minimum temperature (℃), daily average temperature (℃), daily precipitation (mm) and daily average wind speed (M/s) observed by 186 ground observation meteorological stations in the Huang-Huai-Hai region from 1960 to 2019 (Fig. 1). Geographic information data include elevation DEM data (resolution of 1 km × 1 km) and land use data in the study area, which are from the resource and environmental science and data center of Chinese Academy of Sciences (http://www.resdc.cn). Crop data, including peanut sowing area and yield data, are derived from the statistical yearbooks of provinces and cities in the study area and China Agricultural Technology Network (http://www.cast.net.cn).

### Data processing

#### Meteorological data processing

Anusplin software is a tool to interpolate multivariate data based on ordinary thin disks and local thin disk spline functions, enabling the introduction of covariates for simultaneous spatial interpolation of multiple surfaces, suitable for meteorological data time series^29^. First, the Anusplin software is used to spatially interpolate the meteorological data and suitability data of the peanut growing season (April to September) from 1960 to 2019 based on the elevation data with a resolution of 1 km × 1 km. The Inverse Distance Weight (IDW) interpolation can make the meteorological data after Anusplin interpolation maintain consistency with the original data, and is able to improve the interpolation accuracy. Finally, the meteorological and suitability data set with a resolution of 1 km × 1 km is obtained. ArcGIS and MATLAB software were used to count the median of regional meteorological factors in agricultural fields of different cities (counties), and the meteorological factors and suitability of different periods of peanut growth season in each city (county) were obtained.

#### Yield data processing

Many factors affect crop yield formation, which can be generally divided into three main categories: meteorological conditions, agronomic and technological measures, and stochastic factors. Agricultural technical measures reflect the development level of social production in a certain historical period and become time technology trend output, which is referred to as trend output for short, and meteorological production reflects short period yield components that are affected by meteorological elements. Stochastic factors account for a small proportion and are often ignored in actual calculations^30^. The specific calculation is as follows:

1 $$Y={Y}_{t}+{Y}_{w}$$

where Y is the actual yield (single production) of the crop, Y_t_ is the trend yield, and Y_w_ is the meteorological yield.

In this paper, a straight-line sliding average method is used to simulate the trend yield. The straight-line sliding average method is a very commonly used method to model yield, and it considers the change in the time series of yield within a certain stage as a linear function, showing a straight line, as the stage continuously slides, the straight line continuously changes the position, and the backward slip reflects the continuous change in the evolution trend of the yield history^31^. The regression models in each stage are obtained in turn, and the mean value of each linear sliding regression simulation value at each time point is taken as its trend yield value. The linear trend equation at some stage is:

2 $${Y}_{i}\left(t\right)={a}_{i}+{b}_{i}t$$

where i = n-k + 1, is the number of equations; k is the sliding step; n is the number of sample sequences; t is the time serial number. Y_i_(t) is the function value of each equation at point t. there are q function values at point t. the number of q is related to n and k. Calculate the average value of each function value at each point:

3 $$\overline{{Y }_{i}(t)}=\frac{1}{q}\sum_{j=1}^{q}{Y}_{i}\left(t\right)$$

Connecting the $\overline{{Y }_{i}(t)}$ value of each point can represent the historical evolution trend of production. Its characteristics depend on the value of k. Only when k is large enough, the trend yield can eliminate the influence of short cycle fluctuation. After comparison and considering the length of yield series, k is taken as 5 in this paper.

After the trend yield is obtained, the meteorological yield is calculated using Eq. (1), then the relative meteorological production is

4 $${Y}_{r}=\frac{{Y}_{w}}{{Y}_{t}}$$

The relative meteorological yield shows that the relative variability of yield fluctuation deviating from the trend, that is, the amplitude of yield fluctuation, is not affected by time and space, and is comparable. However, when the value is negative, it indicates that the meteorological conditions are unfavorable to the overall crop production, and the crop yield reduction, that is, the yield reduction rate^32^.

#### Characteristics of spatial and temporal distribution of climatic resources in the Huang-Huai-Hai region

Collect meteorological resource data from 1960 to 2019. Taking 1960–1989 as the first three decades of the study and 1990–2019 as the last three decades, the climatic resource changes of peanut growth in the Huang-Huai-Hai region are analyzed by interpolation of heat resources (average temperature), water resources (precipitation) and light resources (sunshine hours) in the study area in two periods combined with topographic factors.

#### Establishment of suitability model

According to the definition of phenological time and growth period of peanut planting practice in the Huang-Huai-Hai region, the growth season of peanut is divided into three growth periods and five growth stages (Table 1). Temperature, precipitation and sunshine hours are the necessary meteorological factors to determine the normal development of peanut. Therefore, combined with climatic resources in the study area, temperature, precipitation and sunshine suitability model was introduced to quantitatively analyze the suitability of peanut planting.

**Table 1:** Division of peanut growth periods.

Growth periods	Growth stages	Features
Pre reproductive period	Emergence stage	From sowing to 50% of the seedlings are unearthed and the first true leaf (compound leaf) is expanded
	Seedling stage	From 50% seed emergence to 50% plant first flower opening
Mid reproductive period	Flowering and needling stage	From 50% of the plants began to blossom to 50% of the plants appeared chicken head like young fruit
Last reproductive period	Pod setting stage	From 50% of plants with chicken head like young fruit to 50% of plants with full fruit
	Full fruit stage	From 50% plants with full fruit to full and mature pods

#### Temperature suitability model

Temperature is a very important factor in the growth period of peanut, and the change of temperature in different growth periods will have a great influence on the yield and quality of peanut. As a warm-loving crop, accumulated temperature plays a decisive role in the budding condition and nutrient growth stage of peanut. Temperature determines the quality of fruit and the final yield of peanut. Beta function^33^ is used to calculate temperature suitability, which is universal for crop-temperature relationship. The specific calculation is as follows:

5 $${F}_{i}\left(t\right)=\frac{(t-{t}_{1}){({t}_{h}-t)}^{B}}{({t}_{0}-{t}_{1}){({t}_{h}-{t}_{0})}^{B}}$$

where the value of B is shown in

6 $$B=\frac{{t}_{h}-{t}_{0}}{{t}_{0}-{t}_{1}}$$

where F_i_(t) is the temperature suitability of a certain growth period; t is the daily average temperature of peanut at a certain development stage; t_1_, t_h_ and t_0_ are the lower limit temperature, upper limit temperature and appropriate temperature required for each growth period of peanut. Refer to the corresponding index system and combined with the peanut production practice in Huang-Huai-Hai region^34–36^, determine the three base point temperature of peanut in each growth period, as shown in the Table 2.

**Table 2:** Three fundamental points temperature and crop coefficient of peanut at each growth stage in the study area.

Growth periods	Lower limit temperature (t_1_)	Appropriate temperature (t_0_)	Upper limit temperature (t_h_)	Crop coefficient (K_c_)
Pre reproductive period	12	22.5	37	0.50
Mid reproductive period	19	27.5	37	1.15
Last reproductive period	15	25	39	0.60

#### Precipitation suitability model

Peanut has a long growth period, which is nearly half a year. Insufficient or excessive water during the growth period has a great impact on the growth and development, pod yield and quality of peanut. Combined with the actual situation of Huang-Huai-Hai region and peanut precipitation / water demand index, the water suitability function is determined and calculated as follows:

7 $${\text{F}}_{{\text{i}}} \left( {\text{r}} \right) = \left\{ {\begin{array}{*{20}l} {\frac{{\text{r}}}{{0.9{\text{ET}}_{{\text{c}}} }}} \hfill & {r < 0.9E{\text{T}}_{{\text{c}}} } \hfill \\ 1 \hfill & {0.9E{\text{T}}_{{\text{c}}} \le r \le 1.2E{\text{T}}_{{\text{c}}} } \hfill \\ {\frac{{1.2{\text{ET}}_{{\text{c}}} }}{{\text{r}}}} \hfill & {r > 1.2E{\text{T}}_{{\text{c}}} } \hfill \\ \end{array} } \right.$$

where F_i_(r) is the water suitability of a certain growth period; r is the accumulated precipitation of peanut in a certain development period; ET_c_ is the water demand of peanut in each growth period.

8 $${\mathrm{ET}}_{\mathrm{c}}={\mathrm{K}}_{\mathrm{c}}\cdot {\mathrm{ET}}_{0}$$

where K_c_ is the peanut crop coefficient (Table 2) and ET_0_ is the crop reference evapotranspiration, which is calculated by the Penman Monteith method recommended by the international food and Agriculture Organization (FAO).

#### Sunshine suitability model

Sunshine hours are an important condition for photosynthesis. The "light compensation point" and "light saturation point" of peanut are relatively high, and more sunshine hours are required for photosynthesis. Under certain conditions of water, temperature and carbon dioxide, photosynthesis increases or decreases with the increase or decrease of light. Relevant studies show that when the sunshine hours reach more than 55% of the available sunshine hours, the crops reach the appropriate state to reflect the light^37^. The following formula is used to calculate the sunshine suitability of peanut in each growth period.

9 $${\mathrm{F}}_{\mathrm{i}}\left(\mathrm{s}\right)=\left\{\begin{array}{l}\frac{\mathrm{S}}{{\mathrm{S}}_{0}} \quad S<{\mathrm{S}}_{0}\\ 1 \quad S>{\mathrm{S}}_{0}\end{array}\right.$$

where Fi(s) is the sunshine suitability of peanut in a certain development period, S is the actual sunshine hours in a certain growth period, S_0_ is 55% of the sunshine hours (L_0_), and the calculation method of L_0_ refers to the following formula.

10 $${\mathrm{L}}_{0}=\frac{2\mathrm{t}}{15}$$

11 $$\mathrm{sin}\frac{\mathrm{t}}{2}=\sqrt{\frac{\mathrm{sin}(45^\circ -\frac{\mathrm{\varnothing }-\updelta -\upgamma }{2})\times \mathrm{sin}(45^\circ +\frac{\mathrm{\varnothing }-\updelta -\upgamma }{2})}{\mathrm{cos\varnothing }\times \mathrm{cos\delta }}}$$

where Φ is the geographic latitude, δ is the declination, γ is the astronomical refraction, t is the angle.

#### Comprehensive suitability model

Peanut has different needs for meteorological elements such as temperature, sunshine and precipitation in different growth periods. In order to analyze the impact of meteorological factors in different growth periods on yield, correlation analysis was conducted between the suitability of temperature, precipitation and sunshine in each growth period and the relative meteorological yield of peanut, and the correlation coefficient of each growth period divided by the sum of the correlation coefficients of the whole growth period was used as the weight coefficient of the suitability of temperature, precipitation and sunshine in each growth period (Table 3). The climatic suitability of each single element in peanut growing season is calculated by using formulas (12) and (13):

**Table 3:** The weight coefficients of climatic suitability at each growth stage.

Growth period	Temperature suitability	Precipitation suitability	Sunshine suitability
Pre reproductive period	0.32	0.33	0.20
Mid reproductive period	0.28	0.42	0.45
Last reproductive period	0.40	0.25	0.35

12 $$\left\{\begin{array}{c}{\mathrm{b}}_{\mathrm{ti}}=\frac{{\mathrm{a}}_{\mathrm{ti}}}{\sum_{\mathrm{i}=1}^{\mathrm{n}}{\mathrm{a}}_{\mathrm{ti}}}\\ {\mathrm{b}}_{\mathrm{ri}}=\frac{{\mathrm{a}}_{\mathrm{ri}}}{{\sum }_{\mathrm{i}=1}^{\mathrm{n}}{\mathrm{a}}_{\mathrm{ri}}}\\ {\mathrm{b}}_{\mathrm{si}}=\frac{{\mathrm{a}}_{\mathrm{si}}}{{\sum }_{\mathrm{i}=1}^{\mathrm{n}}{\mathrm{a}}_{\mathrm{si}}}\end{array}\right.$$

13 $$\left\{\begin{array}{c}F(t)={\sum }_{\mathrm{i}=1}^{\mathrm{n}}\left[{\mathrm{b}}_{\mathrm{ti}}{\mathrm{F}}_{\mathrm{i}}(\mathrm{t})\right]\\ F(r)={\sum }_{\mathrm{i}=1}^{\mathrm{n}}\left[{\mathrm{b}}_{\mathrm{ri}}{\mathrm{F}}_{\mathrm{i}}(\mathrm{r})\right]\\ F(s)={\sum }_{\mathrm{i}=1}^{\mathrm{n}}\left[{\mathrm{b}}_{\mathrm{si}}{\mathrm{F}}_{\mathrm{i}}(\mathrm{s})\right]\end{array}\right.$$

where b_ti_, b_ri_ and b_si_ are the weight coefficients of temperature, precipitation and sunshine suitability in the i growth period respectively, a_ti_, a_ri_ and a_si_ are the correlation coefficients between temperature, precipitation and sunshine suitability and meteorological impact index of peanut yield in the i growth period respectively, and F(t), F(r) and F(s) are the temperature, precipitation and sunshine suitability in peanut growth season respectively.

Then, the geometric average method is used to obtain the comprehensive suitability of peanut growth season, as shown in formula (14).

14 $$F(S)=\sqrt[3]{F(t)\times F(r)\times F(s)}$$

#### Verification of climatic zoning results

##### Drought and flood disaster index

On the basis of previous studies, in view of the different water demand of peanut in different development stages, this paper adds the water demand of peanut in different development stages as an important index to calculate, and constructs a standardized precipitation crop water demand index (SPRI) that can comprehensively characterize the drought and flood situation of peanut, so as to judge and analyze the occurrence of drought and flood disasters of peanut.

Step 1: calculate the difference D between precipitation and crop water demand at each development stage

15 $${D}_{i}={P}_{i}-{ET}_{ci}$$

where P_i_ is the precipitation in the i development period (mm), and ET_ci_ is the crop water demand in the i development period (mm).

Step 2: normalize the data sequence.

Since there are negative values in the original sequence, it is necessary to normalize the data when calculating the standardized precipitation crop water demand index. The normalized value is the SPRI value. The normalization method and drought and flood classification are consistent with SPEI index^38–40^.

##### Chilling injury index

Based on the results of previous studies^41^, the abnormal percentage of caloric index was selected as the index of low-temperature chilling injury of peanut to judge and analyze the occurrence of chilling injury in different growth stages. The specific calculation process and formula are as follows:

Step 1: calculate the caloric index of different development stages.

Combined with the growth and development characteristics of peanut and considering the appropriate temperature, lower limit temperature and upper limit temperature at different growth stages of peanut, the caloric index can reflect the response of crops to environmental heat conditions. The average value of daily heat index is taken as the heat index of growth stage to reflect the influence of heat conditions in different growth stages on crop growth and development. Refer to formulas (5) and (6) to calculate the heat index F_i_(t) at different development stages.

Step 2: calculate the percentage of heat index anomaly

16 $${I}_{ci}=\frac{{F}_{i}(t)-\overline{{F }_{i}(t)}}{\overline{{F }_{i}(t)}}\times 100\%$$

where Ici is the Chilling injury index of stage i, Fi(t) is the heat index of stage i, and $\overline{{F }_{i}(t)}$ is the average value of the heat index of stage i over the years.

##### Heat injury index

Based on the results of previous studies^42^, taking the average temperature of 26 °C, 30 °C and 28 °C and the daily maximum temperature of 35 °C, 35 °C and 37 °C as the critical temperature index to identify the heat damage of peanut in three growth stages, if this condition is met and lasts for more than 3 days, it will be recorded as a high temperature event.

##### Disaster frequency

Disaster frequency (P_i_) is defined as the ratio of the number of years of disaster at a certain station to the total number of years in the study period^43^, which is calculated by formula (17).

17 $${P}_{i}=\frac{n}{N}\times 100\%$$

where n is the number of years of disaster events to some extent at a certain growth period at a certain station, and N is the total number of years.

## Results

### Distribution and comparison of heat, water and light resources in two climatic years in the Huang-Huai-Hai region

#### Heat resources

**Figure 2:**
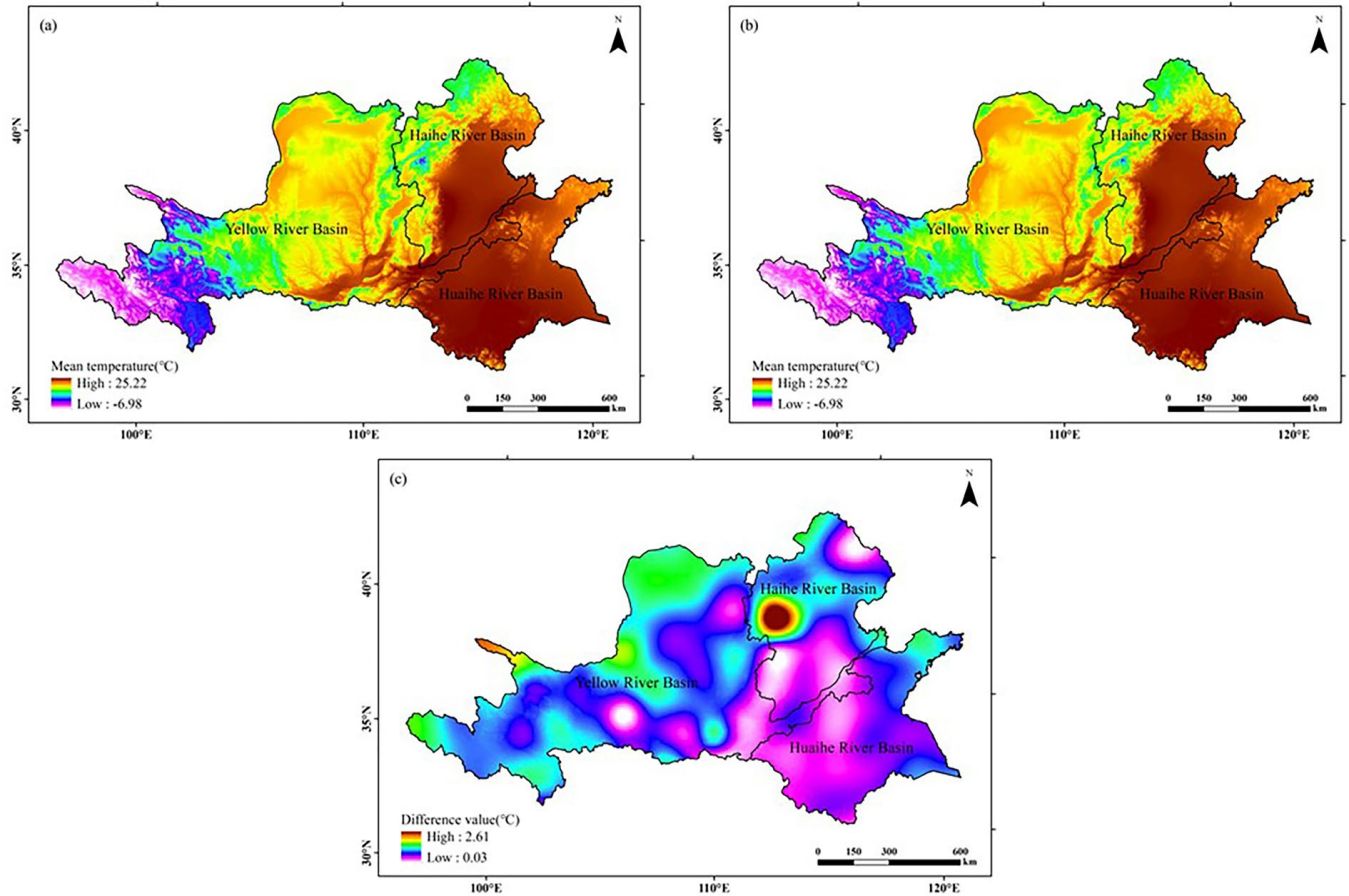
Comparison of average temperature distribution in peanut growing season in the Huang-Huai-Hai region in two climatic years: (**a**) 1960–1989; (**b**) 1990–2019; (**c**) (**b**)–(**a**). The figure was made in the ArcGIS 10.2 platform (https://www.esri.com/en-us/home).

As can be seen from Fig. 2, the mean peanut growing season temperatures in the Huang-Huai-Hai region spatially exhibited a distribution characteristic of high in the east and low in the west in both climatic years. Among them, the high value region appears in the majority of the Huaihe River Basin and the east of the Haihe River Basin, and the low value region appears in the west of the Yellow River Basin. Analyzing from the chronological changes, compared with those in 1960–1989 (former 30a), the average temperature in the whole region of the study area increased to some extent in 1990–2019 (latter 30a), with the lowest temperature increasing by 0.03 °C and the highest temperature increasing by 2.61 °C, appearing in the Midwest of the sea River Basin.

#### Water resources

**Figure 3:**
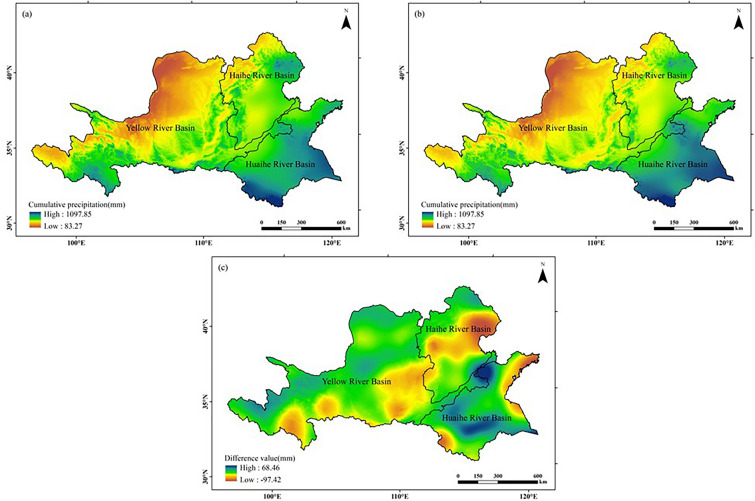
Comparison of cumulative precipitation distribution during peanut growing seasons in the Huang-Huai-Hai region in two climatic years: (**a**) 1960–1989, (**b**) 1990–2019, (**c**) (**b**)–(**a**). The figure was made in the ArcGIS 10.2 platform (https://www.esri.com/en-us/home).

As can be seen from Fig. 3, the spatial distribution of cumulative precipitation during the peanut growing season in the Huang-Huai-Hai region exhibited a decreasing trend from southeast to northwest in both climatic years. Regions with high values of precipitation were concentrated to the south and east of the Huaihe River Basin, and regions with low values were concentrated to the north of the Yellow River Basin. Analyzing the chronological changes, compared with those in 1960–1989 (former 30a), about 60.44% of the study area showed a decrease in cumulative precipitation in 1990–2019 (latter 30a), which was mainly distributed in the south of the Yellow River Basin, the middle and south of the Haihe River Basin and the west and south of the Huaihe River Basin, with a maximum decrease of 97.42 mm.

#### Light resources

**Figure 4:**
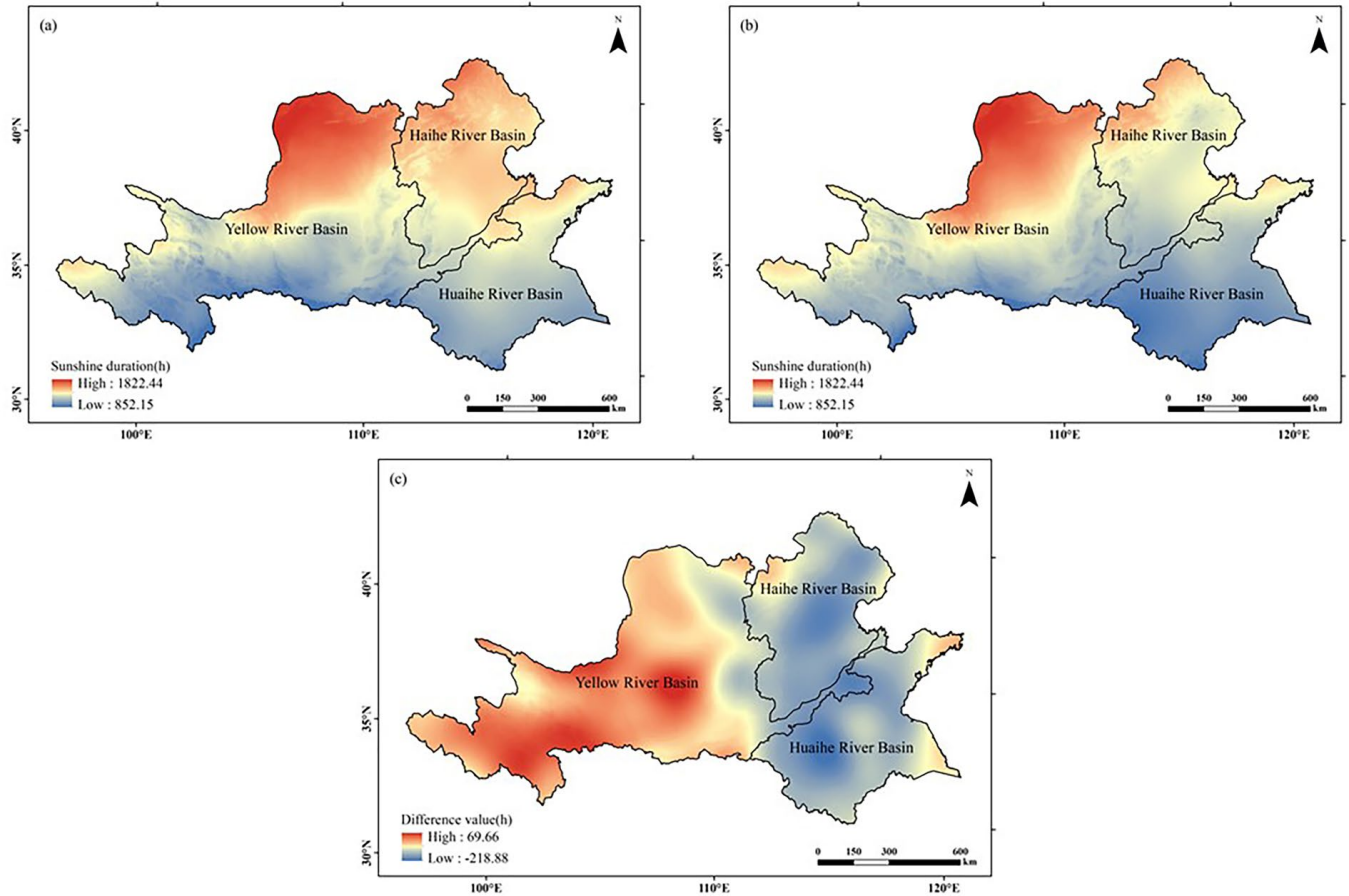
Comparison of the distribution of seasonal sunshine duration for peanut growth in the Huang-Huai-Hai region during two climatic years: (**a**) 1960–1989, (**b**) 1990–2019, (**c**) (**b**)–(**a**). The figure was made in the ArcGIS 10.2 platform (https://www.esri.com/en-us/home).

As can be seen in Fig. 4, the number of peanut growing quarterly sunshine in the Huang-Huai-Hai region spatially exhibited a low north to south distribution in both climatic years. The high value area of sunshine duration mainly appears in the northern Yellow River Basin and the Haihe River Basin, and the low value area mainly occurs in the southern Huai River Basin. Analyzing from the chronological change, compared with those in 1960–1989 (the former 30a), the number of sunshine hours increased in the west and decreased in the east of the study area in 1990–2019 (the latter 30a), where the decrease in sunshine hours was obvious in the Haihe and Huai River Basin, up to 218.88 h.

### Change trend of peanut climatic suitability in Huang-Huai-Hai Region from 1960 to 2019

**Figure 5:**
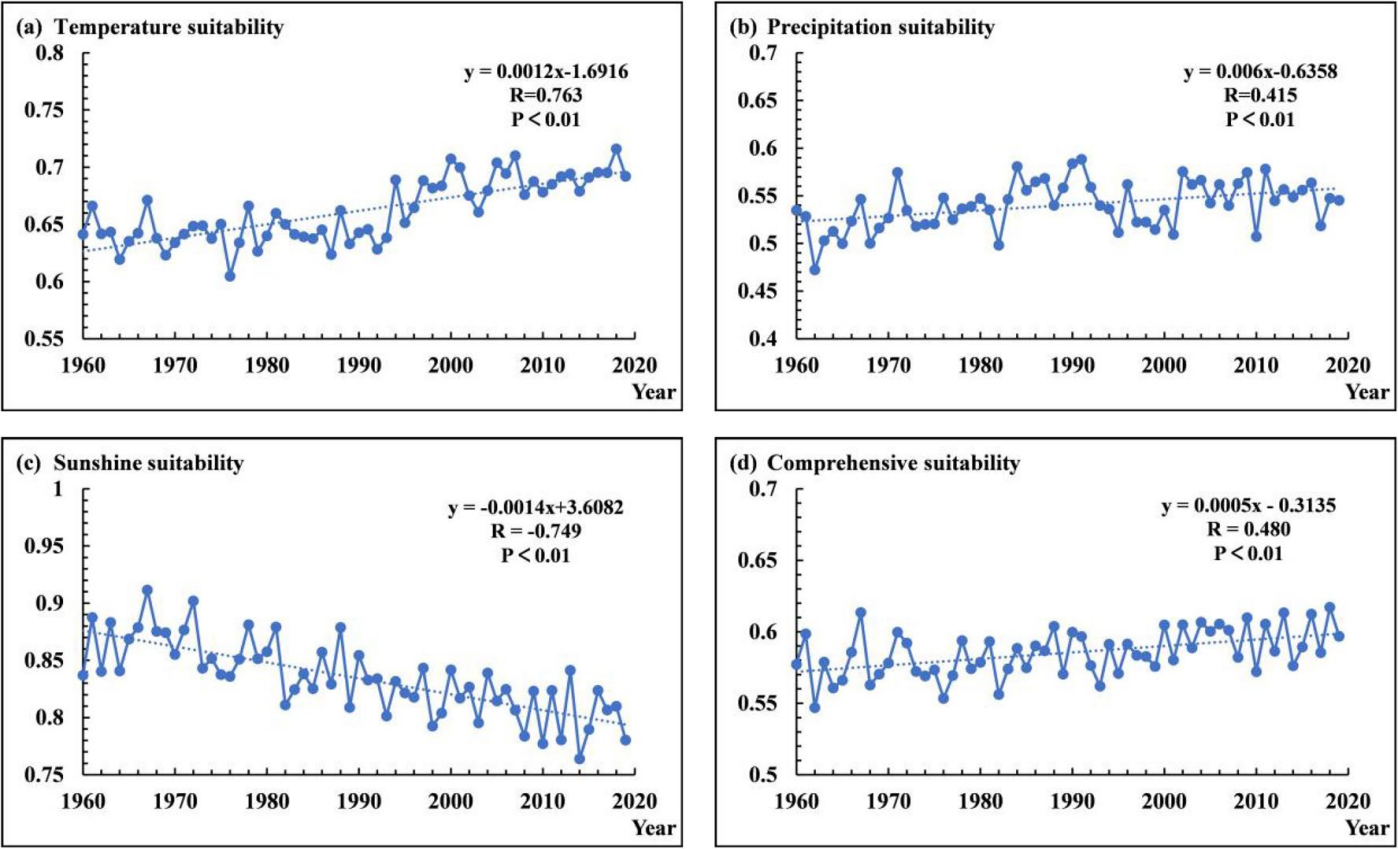
Change trend of peanut climatic suitability in Huang-Huai-Hai Region from 1960 to 2019: (**a**) Temperature suitability, (**b**) Precipitation suitability, (**c**) Sunshine suitability, (**d**) Comprehensive suitability.

The Fig. 5 shows the interannual change of suitability of peanut in the whole growth period from 1960 to 2019 in the study area. From the single factor suitability, the linear increase trend of temperature suitability and precipitation suitability is significant, and the linear decrease trend of sunshine suitability is significant (all three factors passed the 0.01 significance test).At the same time, the linear increasing trend of comprehensive climatic suitability in the whole growth period is significant (passed the 0.01 significance test), indicating that the peanut suitability of the Huang-Huai-Hai region increased in response to climate change.

### Comparison of the suitability of peanut across two climatic years in the Huang-Huai-Hai region

#### Temperature suitability

**Figure 6:**
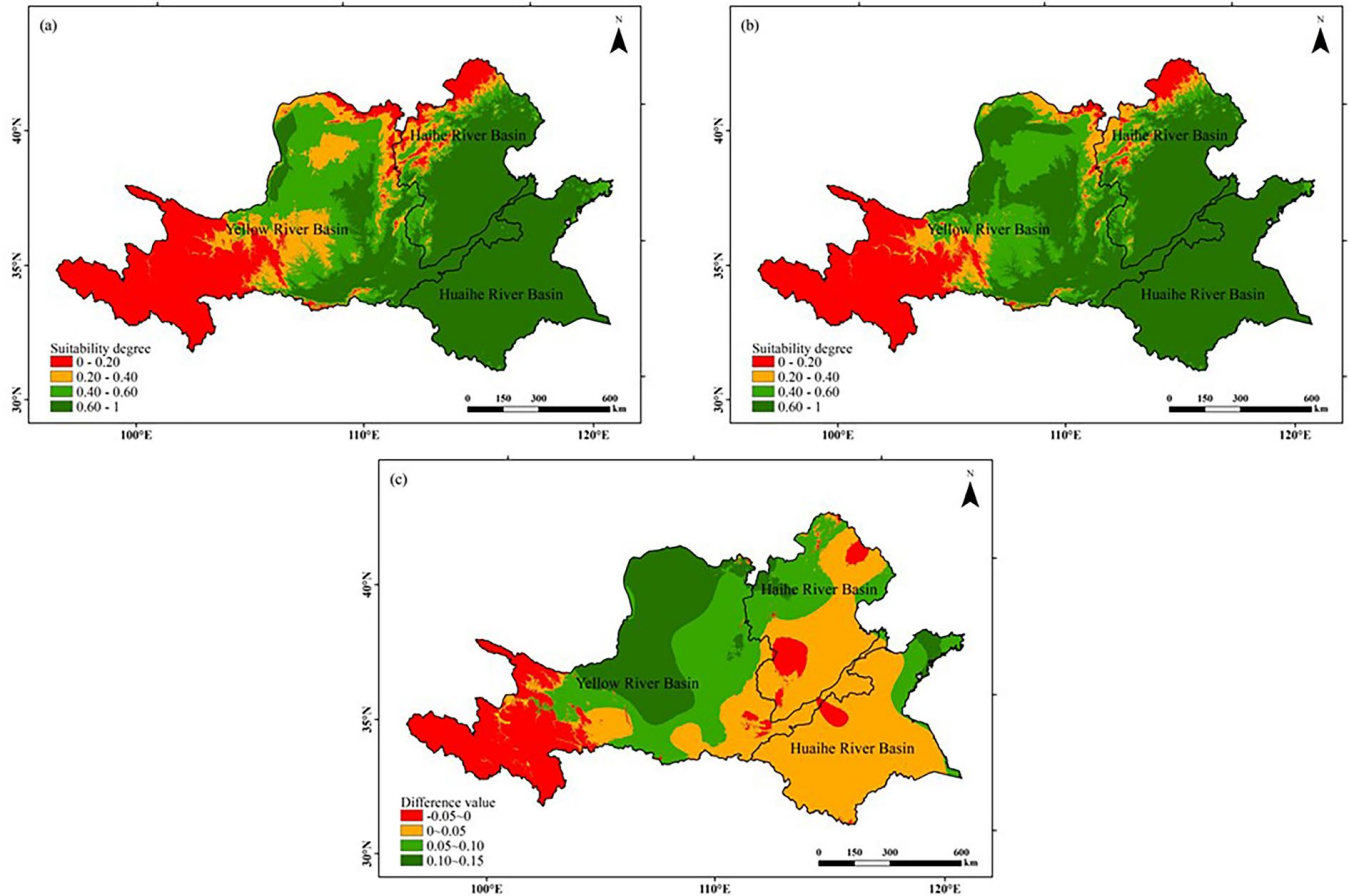
Comparison of the distribution of temperature suitability for peanut growing seasons in the Huang-Huai-Hai region during two climatic years: (**a**) 1960–1989, (**b**) 1990–2019, (**c**) (**b**)–(**a**). The figure was made in the ArcGIS 10.2 platform (https://www.esri.com/en-us/home).

As can be seen from Fig. 6, the temperature suitability during the peanut growing season in the Huang-Huai-Hai region is spatially characterized by low in the west, high in the east, low in the north and high in the south during both climatic years. The high value areas were mainly distributed in the Huaihe River Basin, the Haihe River Basin and the east of the Yellow River Basin, while the low value areas were concentrated in the west and a small part in the east and the north of the Haihe River Basin. Compared with 1960–1989 (former 30a), an increase in the area of high value regions of peanut growth quarterly temperature suitability was observed in the study area in 1990–2019 (latter 30a), mainly in the north central Yellow River Basin, and the area of low value regions was not significantly changed. Overall, about 83.49% of the study area had an increase in regional temperature suitability, and climate warming positively affected peanut growth and development.

#### Precipitation suitability

**Figure 7:**
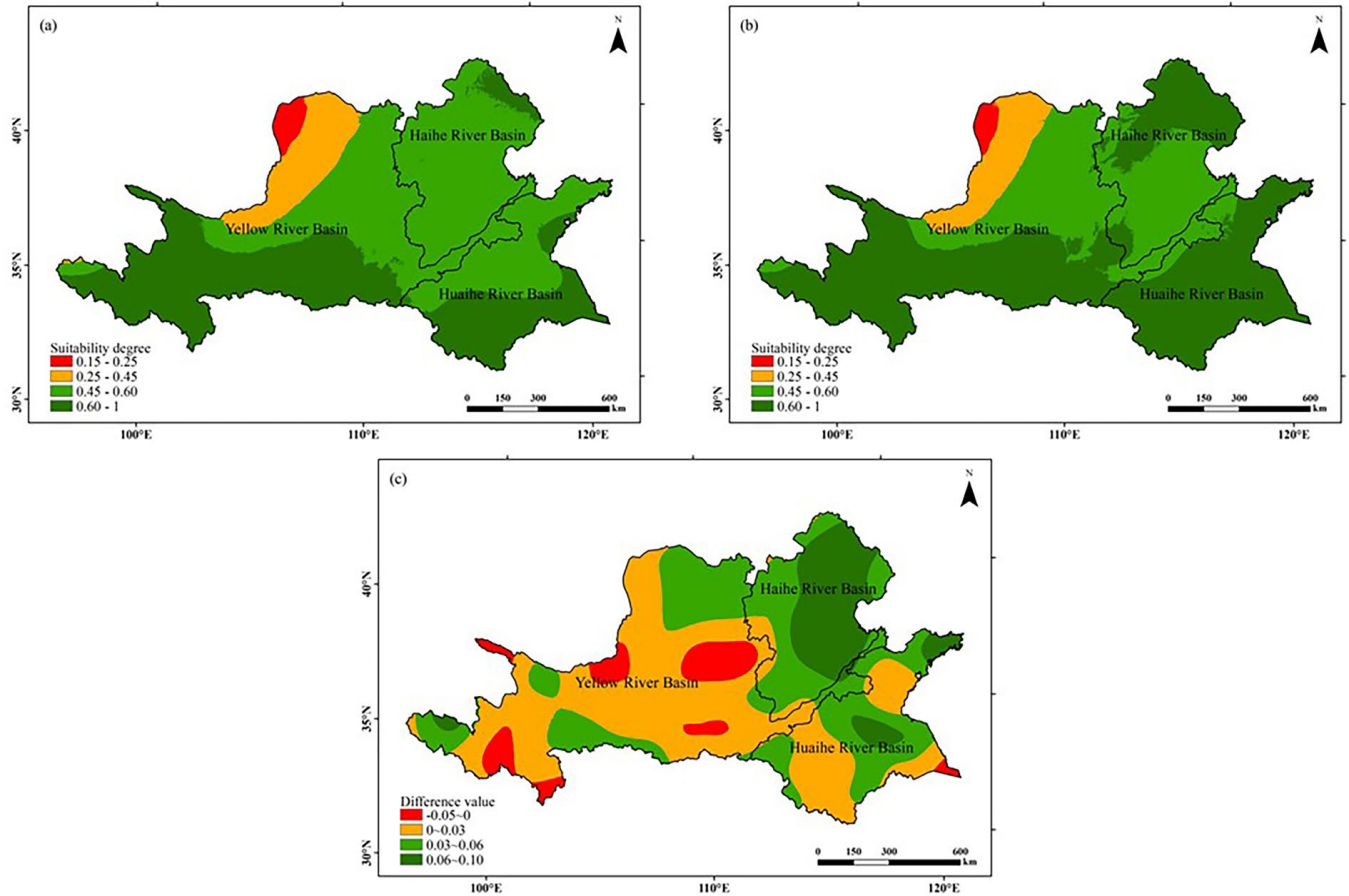
Comparison of distribution of precipitation suitability for peanut growing seasons in the Huang-Huai-Hai region in two climatic years: (**a**) 1960–1989, (**b**) 1990–2019, (**c**) (**b**)–(**a**). The figure was made in the ArcGIS 10.2 platform (https://www.esri.com/en-us/home).

As can be seen from Fig. 7, the precipitation suitability of peanut growing season in the Huang-Huai-Hai region showed a decreasing trend from southeast to northwest during both climatic years. The high value areas appeared in the southern part of the Yellow River Basin, the northern part of the Haihe River Basin and the southern part of the Huaihe River Basin, while the low value areas appeared more concentrated, mainly in the northern part. Analyzing from the chronological changes, the area with high values of precipitation suitability increased by about 20.87% in the study area in 1990–2019 (latter 30 a) compared with 1960–1989 (former 30 a), while the area with low values of precipitation suitability increased mainly in the east, the north and the middle of the Huaihe River Basin, respectively. Overall, with warming, the precipitation suitability increased in 92.37% of the study area, and decreased only in the Yellow River Basin and a very small part of the Huaihe River Basin.

#### Sunshine suitability

**Figure 8:**
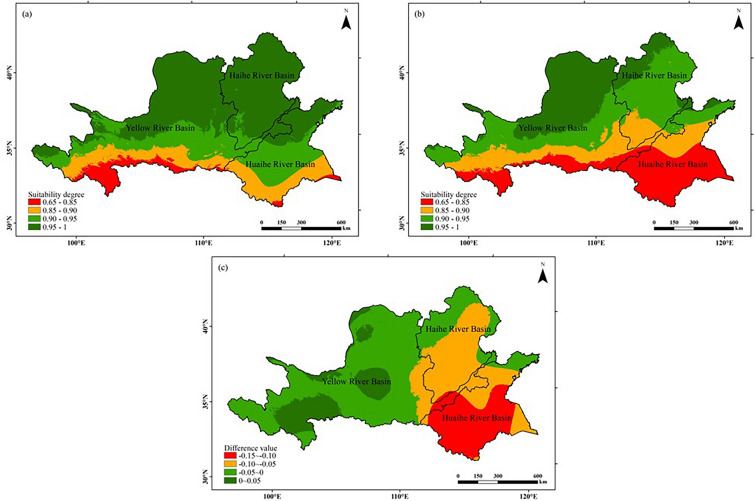
Comparison of the distribution of seasonal sunshine suitability for peanut growth in the Huang-Huai-Hai region during two climatic years: (**a**) 1960–1989, (**b**) 1990–2019, (**c**) (**b**)–(**a**). The figure was made in the ArcGIS 10.2 platform (https://www.esri.com/en-us/home).

As can be seen from Fig. 8, in both climatic years, the seasonal sunshine suitability for peanut growth in the Huang-Huai-Hai region was overall high, with the sunshine suitability in most regions being above 0.90, and only in the southern part of the study area the suitability was low, below 0.85.Analyzing from the chronological changes, the area of the high value area for sunshine suitability in the study area decreased by approximately 27.00% in 1990–2019 (latter 30 a) compared with 1960–1989 (former 30 a), while the areas of high value in the northern, western and southern parts of the Huaihe River Basin largely disappeared, while the area of the low value area in the southern part of the Huaihe River Basin expanded. Overall, due to the impact of climate warming, the sunshine suitability of peanut decreased in 87.33% of the study area.

#### Comprehensive suitability

**Figure 9:**
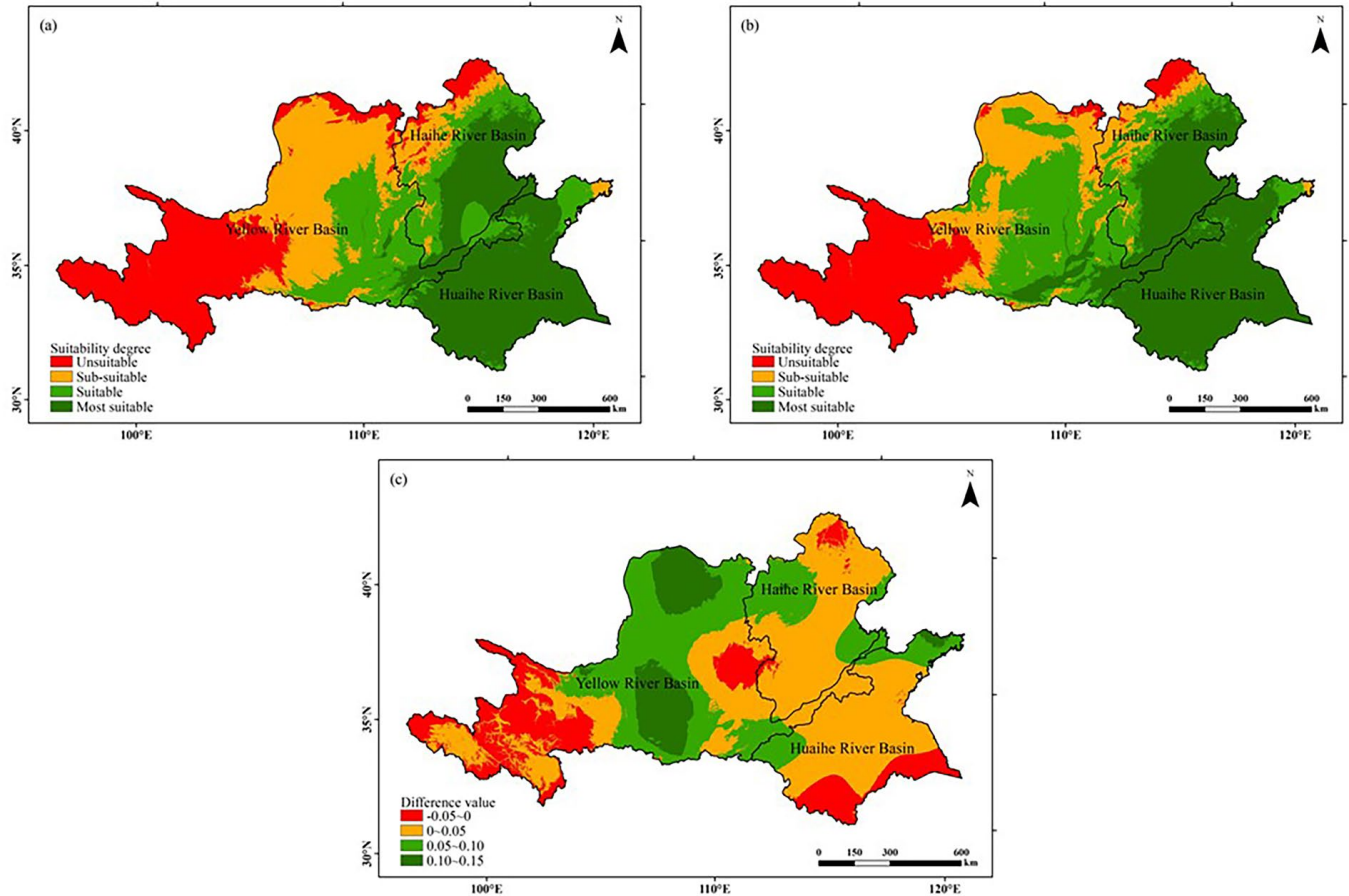
Comparison of comprehensive suitability distribution of peanut growing season in Huang-Huai-Hai region in two climatic years: (**a**) 1960–1989; (**b**) 1990–2019; (**c**) (**b**)–(**a**). The figure was made in the ArcGIS 10.2 platform (https://www.esri.com/en-us/home).

The graph of the climatic suitability of peanut cultivation in the study area was generated using the natural breakpoint method by dividing the integrated suitability of peanut in the study area into four levels: the most suitable, suitable, sub-suitable, and unsuitable (Fig. 9).

Most suitable region (0.60 ≤ S < 1): In 1960–1989 (former 30a), the most suitable planting area for peanut was mainly distributed in the west of the Haihe River Basin and most parts of the Huaihe River Basin, which accounted for approximately 31.31% of the total area in the study area. In 1990–2019 (latter 30a), the most suitable planting area for peanut was expanded before and mainly concentrated in the north of the Huaihe River Basin, the south of the Haihe River Basin and the southwest of the Yellow River Basin, and the increased area accounted for approximately 5.94% of the study area. The growth season average temperature of the most suitable growing region for peanut is 20 ~ 25 °C, the cumulative precipitation is 600 ~ 1000 mm, the number of sunshine hours is 800 ~ 1400 h, and the heat, water and light resources are all suitable for peanut growth and development, which is the most ideal region for peanut cultivation.

Suitable region (0.40 ≤ S < 0.60): In 1960–1989 (former 30a), peanut suitable planting areas were widely distributed, mainly in the middle east of the Yellow River Basin, the middle west of the Haihe River Basin and the northern Huaihe River Basin, which cover approximately 20.88% of the total study area. In 1990–2019 (latter 30a), the range of peanut suitable planting area shifted to the west of the study area, and the area has increased, accounting for 25.09% of the total area in the study area. The climatic conditions in peanut suitable growing areas for peanut production were overall slightly less suitable than those in the most suitable areas, but were able to meet the needs of normal growth and development of peanut, and suitable for peanut growth.

Sub-suitable regions (0.20 ≤ S < 0.40): In 1960–1989 (former 30a), peanut sub-suitable planting areas were mainly distributed in the central and northern Yellow River Basin, the northern Haihe River Basin and the northeast corner of the Huaihe River Basin, and the area occupied approximately 24.15% of the total study area. In 1990–2019 (latter 30a), the area of the sub-suitable planting area of peanut decreased compared with that before, and overall shifted to the northwest. The comprehensive suitability of peanut sub-planting regions was poor, and the reasons for poor comprehensive suitability were different in different regions.

Unsuitable regions (S < 0.20): In 1960–1989 (former 30a), the distribution of peanut unsuitable growing areas was large, which cover approximately 23.66% of the total area in the study area and mainly exists in the west and north of the study area, with the Yellow River Basin dominated by the west. A 3.59% decrease in peanut unsuitable growing areas occurred in 1990–2019 (latter 30a), mainly in the north and west of the Yellow River Basin. The climatic conditions of not suitable regions are extremely low suitable for peanut cultivation, and inhospitable for peanut cultivation.

### Verification of peanut climatic zoning results in Huang-Huai-Hai region

To verify the rationality of the results of the evaluation of the climatic suitability of peanut in the Huang-Huai-Hai region, two aspects are considered in this paper. The former is the occurrence of peanut disasters in the Huang-Huai-Hai region, and the latter is the relative meteorological production change and multi-year average per unit yield of peanut in the Huang Huai Hai region.

#### Drought and waterlogging disaster verification

**Figure 10:**
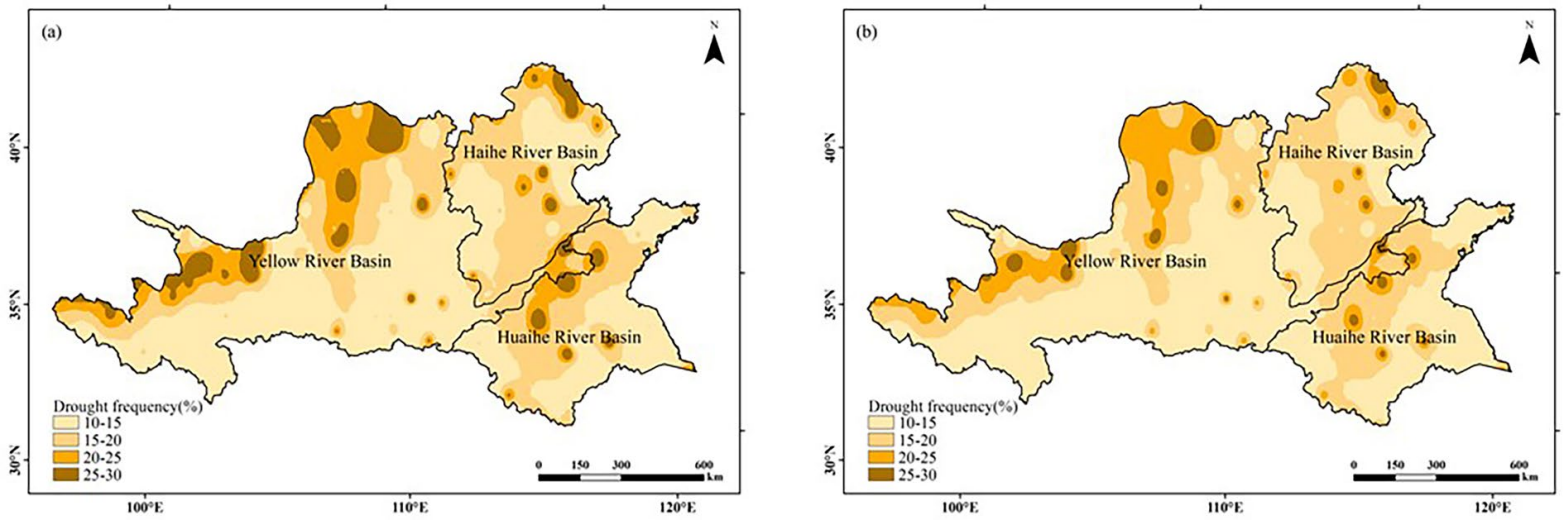
Drought frequency of peanut growing season in Huang-Huai-Hai region in two climatic years: (**a**) 1960–1989; (**b**) 1990–2019. The figure was made in the ArcGIS 10.2 platform (https://www.esri.com/en-us/home).

**Figure 11:**
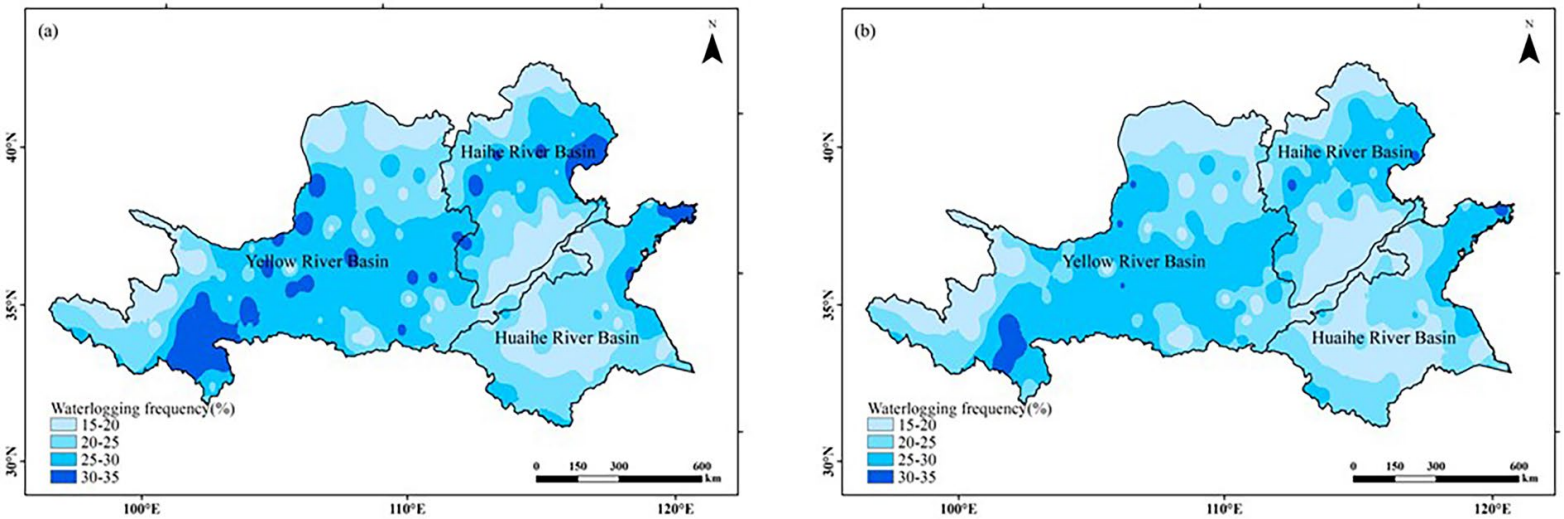
Waterlogging frequency of peanut growing season in the Huang-Huai-Hai region in two climatic years: (**a**) 1960–1989; (**b**) 1990–2019. The figure was made in the ArcGIS 10.2 platform (https://www.esri.com/en-us/home).

The overall distribution of drought and waterlogging disasters within the study area was similar in both climatic ages (Figs. 10, 11). Drought prone areas are concentrated in the northwest and north of the Yellow River Basin, the north of the Haihe River Basin and the north of the Huaihe River Basin, and waterlogging prone areas are concentrated in the southwest and middle of the Yellow River Basin, the east of the Huaihe River Basin and the north of the Huaihe River Basin. With climate change, the frequency of drought and waterlogging disasters in the study area has both decreased. Because drought and waterlogging disasters occur frequently in the west and north of the Yellow River Basin and the north of the Haihe River Basin, it is not suitable for peanut planting. The frequency of drought and flood disasters in the south of Haihe River Basin and most areas of Huaihe River Basin is low, so it is suitable for peanut planting. The verification results are basically consistent with Fig. 9.

#### Chilling injury and heat injury verification

**Figure 12:**
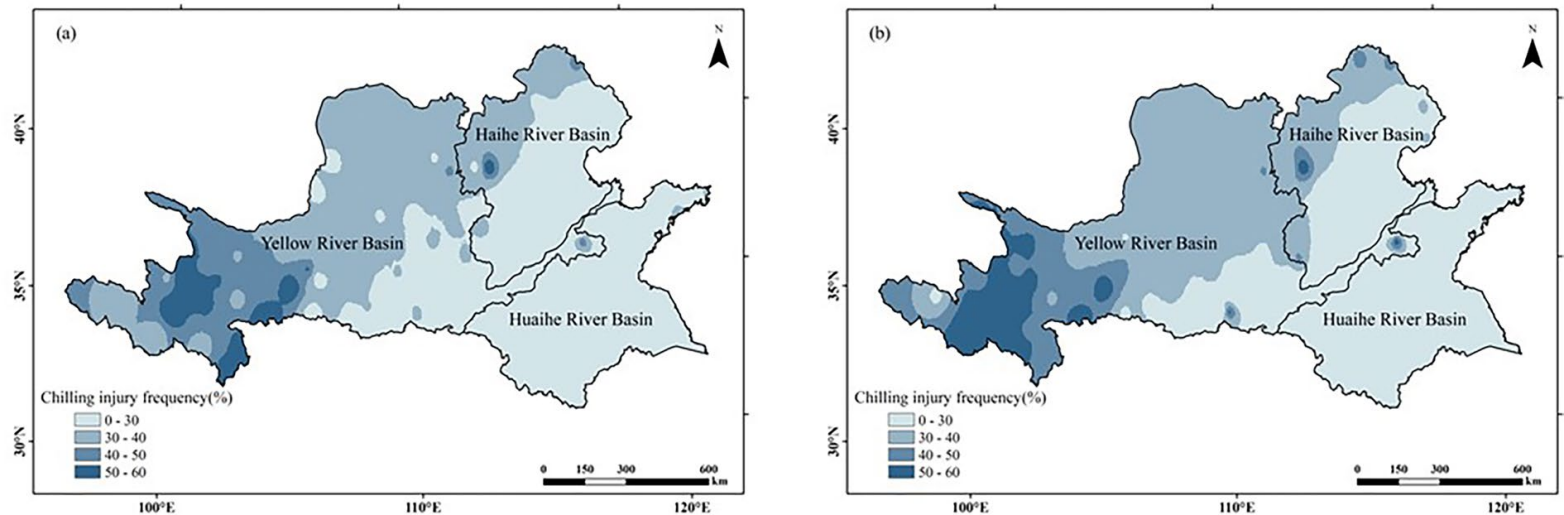
Chilling injury frequency of peanut growing season in Huang-Huai-Hai region in two climatic years: (**a**) 1960–1989; (**b**) 1990–2019. The figure was made in the ArcGIS 10.2 platform (https://www.esri.com/en-us/home).

**Figure 13:**
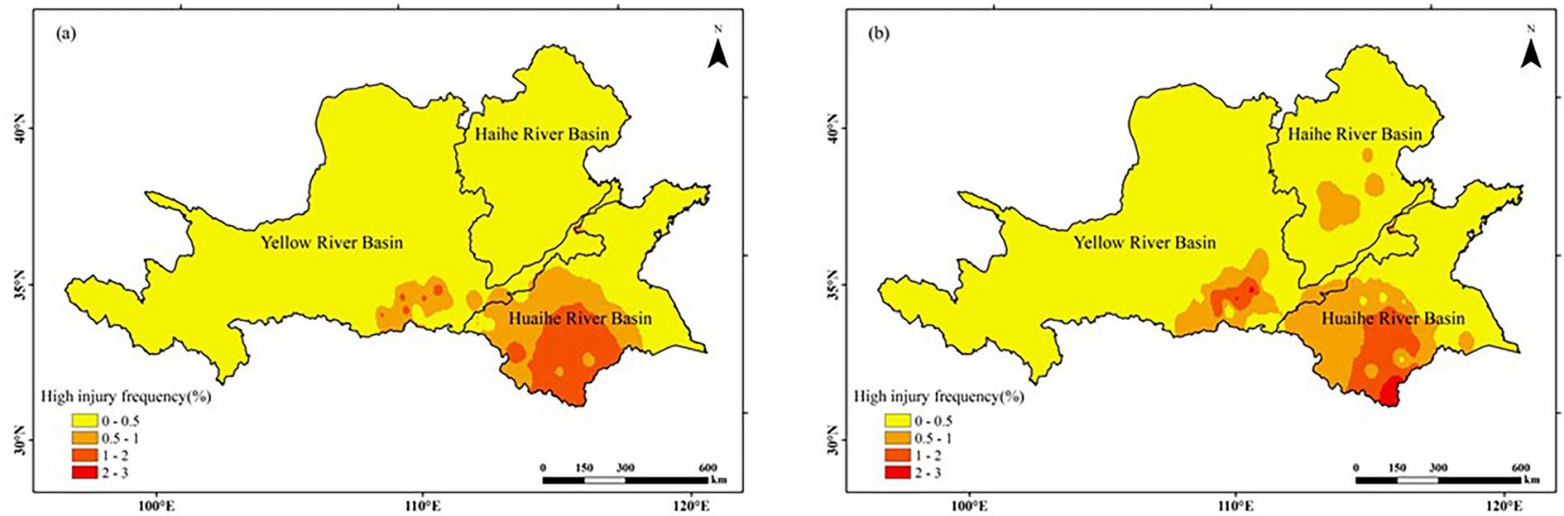
Heat injury frequency of peanut growing season in Huang-Huai-Hai region in two climatic years: (**a**) 1960–1989; (**b**) 1990–2019. The figure was made in the ArcGIS 10.2 platform (https://www.esri.com/en-us/home).

In the two climatic ages, the overall distribution of low-temperature disasters in the study area is similar (Fig. 12). The frequent chilling injury area is mainly concentrated in the west of the Yellow River Basin, which is not suitable for peanut planting. High temperature events are mainly concentrated in the south of the study area (Fig. 13), and the occurrence frequency is low (the maximum is 3%). As a thermophilic crop, heat injury has little impact on the climate suitability of peanut in the study area. The verification results are basically consistent with Fig. 9.

#### Yield verification

**Figure 14:**
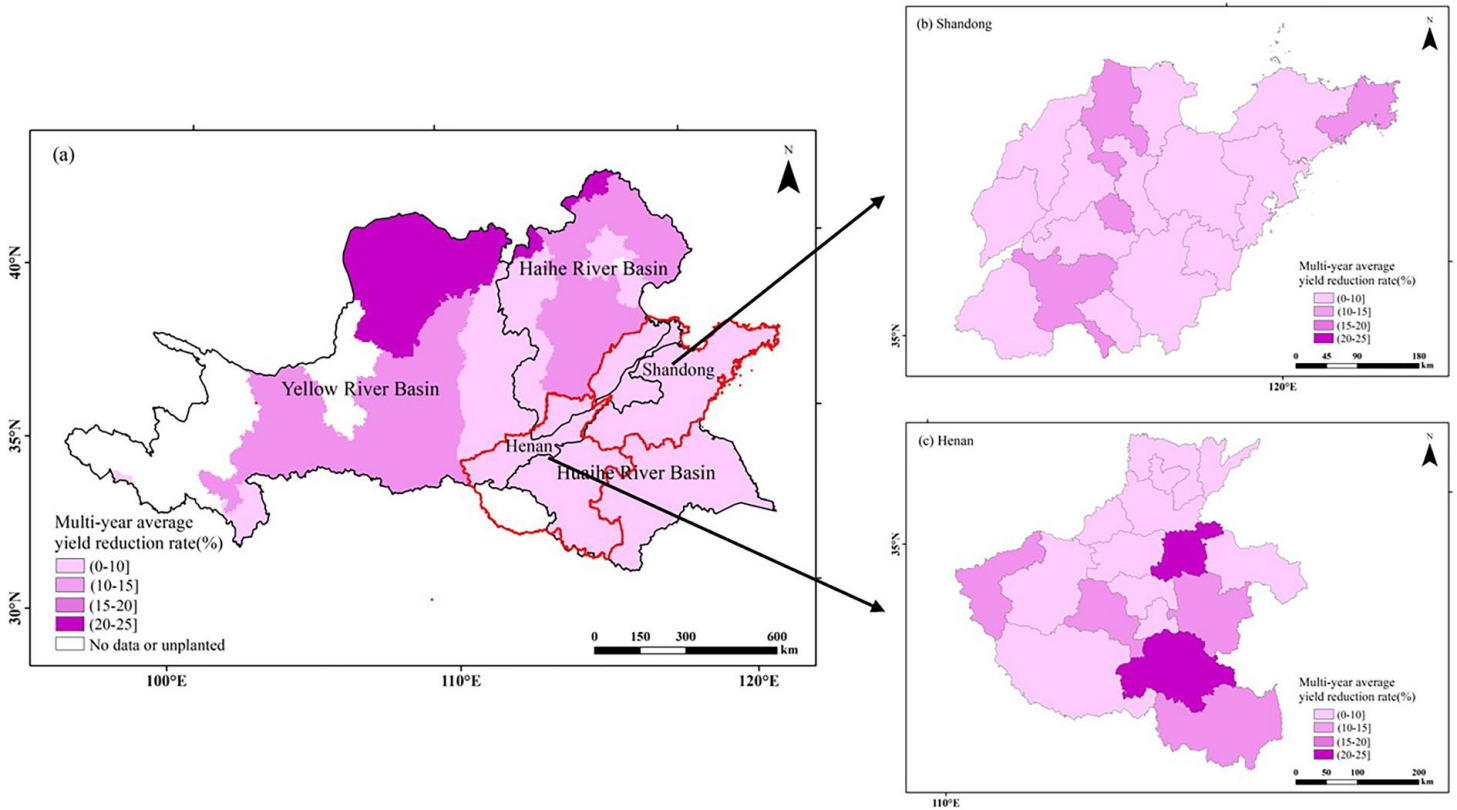
Spatial distribution map of multi-year average yield reduction rate of peanut in Huang-Huai-Hai region: (**a**) Huang-Huai-Hai region; (**b**) Shandong province; (**c**) Henan province. The figure was made in the ArcGIS 10.2 platform (https://www.esri.com/en-us/home).

**Figure 15:**
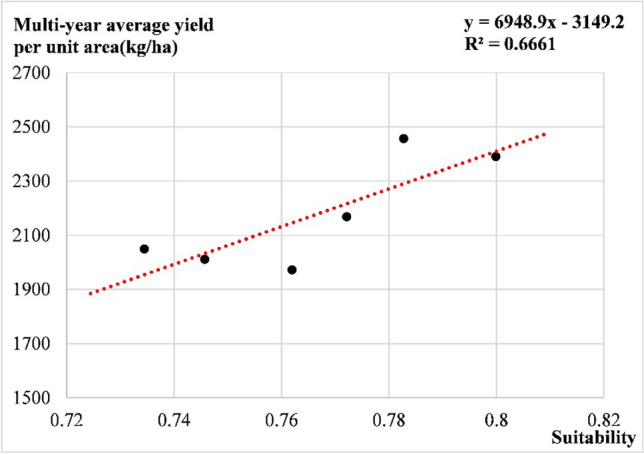
Correlation of multi-year average yield per unit area and comprehensive suitability of peanut in Huang-Huai-Hai region.

According to the multi-year average crop yield reduction rate, spatial distribution map of yield reduction rate in the study area is obtained, and taking Henan and Shandong as examples to make the spatial distribution of municipal yield reduction rate, the results shows that the spatial distributions are consistent with the comprehensive suitability (Fig. 14). In addition, conducting a correlation analysis between multi-year average per unit yield and comprehensive suitability in the study area shows a high correlation (Fig. 15). Thus, it can be concluded that the comprehensive suitability model established in this study can well reflect the suitability of peanut cultivation in the Huang-Huai-Hai region.

## Discussion

The maps of land suitability and crop zones is a complex problem with many contributing factors. Because of climate change, the disturbance of climatic resources can drastically affect crop yields. In a study conducted to evaluate the ecological suitability of peanut in China^26^, the authors concluded that as the suitability grade decreased, the temperature indicator showed a decreasing trend, meanwhile the inflection point of the lowest temperature showed a very significant diminishing tendency, so the temperature was considered to be the most limiting factor in determining the ecological suitability evaluation of peanut. It is also true that temperature is included in many studies as the most influential aspect affecting crop suitability^44^, but these studies have essentially been conducted at the national scale^11^, nevertheless Climatic factors that influence the suitability at the national level may differ from those affecting climate at the regional level. Schewe et al. noted that climate change may significantly exacerbate water scarcity, that reduced rainfall will directly affect agricultural production^45^, he also pointed that dry and waterlogging have also become one of the most severe natural disasters in China. In conclusion, in this study, due to the temperate monsoon climate in the Huang-Huai-Hai region, temperature fluctuates evenly in the peanut growing season so that it can basically meet peanut's temperature demand. Peanut is also very sensitive to water, so precipitation is used to determine whether peanut is short of water. The water sources of peanut from Huang-Huai-Hai region include natural precipitation, groundwater irrigation, and artificial irrigation, whereas it mainly relies on natural precipitation because of the limited availability of groundwater in the area and the lack of irrigation space and uneven distribution of artificial irrigation and precipitation facilities^46^. Therefore, it is reasonable to validate the peanut suitability division in the study area using the frequency of drought and waterlogging.

In this study, we found that different adaptations of peanuts to climate change result in both positive and negative effects on its production. Contrasting the two climatic years, it can be seen that the temperature rose throughout the study area, which allowed 83.49% of the regional temperature appropriateness to increase, indicating that the rising temperature positively affected peanut development. In terms of the duration of sunshine, the number of sunshine hours increased in the Yellow River Basin, while the number of sunshine hours decreased significantly in the Huaihe and Haihe river basins, which led to the basically disappearance of high values of sunshine, and a decrease in the suitability of peanuts in 87.33% of the study area. Precipitation was decreasing in 60.44% of the study areas and even reached 97.42 mm in the middle of the Haihe and Huaihe River Basin, However, due to the extreme concern for peanuts, prefer to choose sandy soil with little moisture and loose aeration, which requires much less water than crops such as maize, wheat and cotton. In the pre reproductive period of peanut, excessive water at sowing will cause erosions, and excessive soil water at the seedling stage will cause poor root growth, yellow leaves, thin plants and poor nitrogen fixation. Therefore, with climate change, the reduce of precipitation increases the suitability of peanut for precipitation in 92.37% of the study area, which directly affects the climatic suitability of peanuts. Altieri et al. proposed some measures to improve suitability, using smart agricultural systems, such as field-scale micro catchment basins or semi-permanent catchment basins, to maintain crop production under climate change^47^. Taking advantage of the calculation of comprehensive suitability, the most suitable and suitable area for peanut cultivation in the Huang-Huai-Hai region in the first climatic year accounted for 52.19% of the whole study area and increasing to 62.34% in the second climatic year, which is a result we wish to see. According to the changing trend of their suitability, we are also able to discover more potential planting areas, and increase in grain yield can be substantially improved by rational planting layout. In less suitable and not suitable regions, it is possible to change their industrial structure or adopt ways such as multiple crop rotations to maximize their spatial availability.

In the context of climate change, we mainly analyze the impact of meteorological factor changes on peanut planting suitability, but rarely mention non meteorological factors which also affect the suitability of crop planting. First, the structure of peanut cultivation is determined by local environmental conditions and socioeconomic factors, if the local government provides a peanut cultivation subsidy, more farmers will go for peanut cultivation, which explains the existence of the large amount of peanut cultivation in many unsuitable areas. Due to the advancement of technology, perfect infrastructure and advanced cultivation technology can also ensure the safety of peanut in the planting process, which is the reason for the difference between the actual planting region and the suitable planting region. Second, peanut phenological variables may also differ among cultivars, as well as the response to climatic suitability, for which a relevant differential analysis may make the zoning results more accurate. However, large-scale climatic suitability evaluation is performed with a focus on climatic indicators. The present study only considers the impact of meteorological factors on peanut planting suitability, and the next research will take into account factors such as soil and planting system. In addition, there is growing interest in the study of climatic suitability zoning of crops in the context of global climate change. In the future research, it is important to refine the analysis of the shortcomings from above as well as to explore the impacts of future scenarios on climatic suitability, in order to provide reasonable layout predictions and provide a scientific basis for insurance development and safe production assurance system construction for disaster weather indexes.

## Conclusions

In order to address the challenges posed by climatic resource change under the background of climate change, this study took peanut in the Huang-Huai-Hai region of China as the research object, determined its climatic zoning index of peanut based on temperature, precipitation, sunshine and comprehensive factors, established the suitability model, and evaluated the climatic suitability of peanut in the Huang-Huai-Hai region, and the climatic suitability evaluation of peanut in the Huang-Huai-Hai region was carried out in two climatic years :1960–1989 and 1990–2019. The main conclusions are as follows.

In terms of climatic resources, both climatic ages within the study area showed a spatial distribution of "The average temperature is higher in the east and lower in the west, the cumulative precipitation decreases from southeast to northwest, and the sunshine hours are higher in the east and lower in the west ".With climate change, the average temperature in the whole study area showed an upward trend, the cumulative precipitation in most areas showed a downward trend, the sunshine hours increase in the west and decrease in the east, and the climate tended to be warm and dry.

In terms of the spatial distribution of climatic suitability during the growing season, the main distribution characteristics of the two climatic ages in the study area were as follows: The temperature suitability is higher in the east and south, lower in the west and north, the precipitation suitability decreases from southeast to northwest, and the sunshine suitability is generally high. With climate change, both temperature suitability and precipitation suitability showed an overall increasing trend, while sunshine suitability decreased greatly.

Based on the comprehensive suitability model, the climatic suitability of peanut for the Huang-Huai-Hai region was divided into the most suitable (0.60 ≤ S < 1), suitable (0.40 ≤ S < 0.60), sub-suitable (0.20 ≤ S < 0.40) and unsuitable (S < 0.20). Among them, the most suitable regions are concentrated in the west of the Haihe River Basin and most parts of the Huaihe River Basin, while the unsuitable regions are concentrated in the west of the Yellow River Basin. With climate change, the most suitable and suitable regions for peanut planting have increased, and the sub-suitable and unsuitable regions have decreased.

The verification results showed that the climatic zoning results had a high correlation with peanut per unit yield and disasters frequency, while also were consistent with the distribution of the dominant peanut producing areas in the Huang-Huai-Hai region^48^.

The disturbance of climatic resources due to climate change affects the regional suitability of crop cultivation, resulting to poor crop growth and development, which in turn affects food security and socioeconomic development. In this study, the climatic suitability evaluation of peanuts in the Huang-Huai-Hai region was carried out to provide references for the production and optimization of planting structure of peanuts in this region.
